# AFP Stimulates Glucose Metabolic Reprogramming Contributing to Hepatocellular Carcinoma Resist Sorafenib Through Activating PI3K/AKT Signalling Pathway

**DOI:** 10.1111/jcmm.71226

**Published:** 2026-06-04

**Authors:** Yuli Zhou, Siren Feng, Yi Chen, Bo Lin, Wei Li, Xu Dong, Kun Liu, Qiushi Yin, Mengsen Li, Mingyue Zhu

**Affiliations:** ^1^ Key Laboratory of Tropical Translational Medicine, Ministry of Education, and Hainan Provincial Key Laboratory of Carcinogenesis and Intervention, School of Basic Medical Sciences Hainan Medical University Haikou Hainan Province P.R. China; ^2^ Department of Medical Oncology, Second Affiliated Hospital Hainan Medical University Haikou Hainan Province P.R. China

**Keywords:** alpha fetoprotein, drug resistance, glucose metabolic reprogramming, hepatocellular carcinoma, PI3K/AKT signalling pathway

## Abstract

Alpha fetoprotein (AFP) regulates glucose metabolism reprogramming (GMR) related to drug resistance of hepatocellular carcinoma (HCC) and remains unclear. This study explores the effect of AFP regulating GMR (Glucose metabolic reprogramming) on the tolerance of HCC cells to sorafenib. Thirty clinical liver cancer samples and multi‐omics databases were collected; the expression of AFP, GMR‐related proteins, pyruvate kinase M2 (PKM2), and the PI3K/AKT signalling pathway‐associated proteins were assessed using immunohistochemistry (IHC) or Western blotting. MTT, cloning assays, flow cytometry, and TUNEL were performed to evaluate the effects of AFP on HCC resistance to sorafenib. Alterations in glucose consumption, lactate dehydrogenase activity, and ATP production were measured. Co‐immunoprecipitation and immunofluorescence experiments were conducted to determine how AFP interacts with PKM2. An in vivo mouse tumour model was used to investigate the restoration of tumorigenesis and development. The results indicated that AFP inhibited sorafenib‐induced apoptosis of HCC cells. AFP activated the PI3K/AKT signalling pathway to promote the GMR‐related protein expression and enzyme activity. Particularly, AFP's interaction with PKM2 stimulated the activity of PKM2 to enhance GMR, contributing to HCC resistance to sorafenib. In vivo experiments demonstrated that inhibition of AFP expression attenuated tumorigenesis and growth, and this effect was restored by overexpression of PKM2; PKM2 played a critical activated role in AFP mediating the GMR in HCC. In conclusion, AFP activates the PI3K/AKT signalling pathway to augment aerobic glycolysis in HCC cells, leading to HCC resistance to sorafenib. Inhibition of AFP expression and targeting of PKM2 may represent a novel approach for clinically reversing sorafenib tolerance in HCC patients.

## 1 Introduction

Hepatocellular carcinoma (HCC) constitutes the substantial majority (75%–85%) of primary liver malignancies and is a significant global problem. HCC is frequently diagnosed at an advanced stage, characterized by a high rate of metastasis, and is associated with a high mortality rate [1, 2]. Treatment options for patients with advanced HCC encompass various modalities such as local ablation, radiotherapy, chemotherapy, and molecular targeted therapy [3]. Sorafenib, a multi‐targeted tyrosine kinase inhibitor, was approved by the U.S. Food and Drug Administration (FDA) for the treatment of advanced HCC in 2005 [4, 5]. Sorafenib prolongs the overall survival of patients with HCC [6], but patients are susceptible to developing resistance within 6 months of treatment [7]. The mechanisms of sorafenib resistance are complex and include activation of the epidermal growth factor receptor (EGFR), c‐Jun, and Akt pathways as well as enhancement of cancer stem cells and epithelial‐mesenchymal transition (EMT). Identifying key targets of sorafenib resistance is essential for the treatment of patients with HCC [8].

Alpha fetoprotein (AFP) belongs to the human serum albumin (HSA) family and is significantly expressed during embryonic development and certain pathological conditions [8, 9, 10]. During the embryonic stage, AFP is sequentially expressed in the extraembryonic visceral yolk sac, fetal liver, intestine and kidneys [11, 12]. Notably, a substantial amount of AFP protein has been detected in the serum of HCC patients, leading to its proposal as a crucial tumour marker for primary liver cancer [13, 14, 15]. Furthermore, AFP exhibits numerous biological functions including crucial involvement in HCC progression, stimulation of HCC cell proliferation and migration, facilitation of immune evasion and inhibition of HCC cell apoptosis [8, 10, 16]. Research has also substantiated the strong association between AFP and multidrug resistance in tumours. For instance, AFP‐positive gastric cancer is more prone to multidrug resistance than typical gastric cancers [16]. Previous investigations conducted by our research team have additionally demonstrated that AFP can induce liver cancer cells to resist all‐trans retinoic acid by activating the PI3K/AKT signalling pathway [17]. However, whether AFP is also involved in sorafenib resistance in HCC remains unclear.

Alterations in cancer cell energy metabolism are considered a prominent characteristic of cancer cells. In the early 20th century, Warburg discovered that tumour cells utilized aerobic glycolysis to metabolize glucose for energy production, a phenomenon known as the Warburg effect [18, 19]. Pyruvate kinase M2 (PKM2) is a key rate‐limiting enzyme in glycolysis, regulates glucose metabolic reprogramming (GMR), which facilitates the Warburg effect and is significantly upregulated in numerous cancer tissues [20, 21, 22]. Extensive research has confirmed the high expression of PKM2 in HCC tissues, establishing its close association with patient prognosis of patients [23, 24]. However, the specific role and underlying mechanism of PKM2 in liver cancer remain unclear. Previous studies have demonstrated that PKM2 can confer drug resistance by modulating metabolic energy balance, and its overexpression has been associated with multidrug resistance in cancers [25, 26, 27]. Notably, prolonged exposure of cancer cells to oxaliplatin has been shown to upregulate PKM2 expression, subsequently leading to drug resistance [28]. Conversely, inhibition of PKM2 expression has been found to reverse resistance to 5‐Fu in colorectal cancer cells [26]. Interestingly, PKM2 has been found to promote sorafenib resistance in HCC cells by increasing glycolytic flux [29].

Recent findings indicated a close association between high PKM2 expression and clinical features such as AFP expression in cancer tissues [30, 31]. Previous research conducted by our laboratory revealed that AFP could hinder the inhibitory effect of PTEN on PI3K/AKT and subsequently activate the PI3K/AKT signalling pathway [32]. Considering the significance of the PI3K/AKT signalling pathway in cellular metabolism, we hypothesized that AFP regulates the expression and activity of PKM2 by activating the PI3K/AKT signalling pathway. The aim of this study was to elucidate the importance of AFP as a key factor in HCC, investigate the interaction between AFP and PKM2‐mediated GMR, and explore the unique molecular mechanism of sorafenib resistance in HCC.

## 2 Materials and Methods

### 2.1 Samples of Human HCC Tissues

This study included patients admitted to the Second Affiliated Hospital of Hainan Medical University between January 2019 and December 2022 for initial HCC excision. Thirty patients who met the eligibility criteria were included in the analysis. Tumour staging was performed using the Barcelona Clinical Liver Cancer (BCLC) staging system [33]. Informed consent was obtained from all participants and the collection of tissue samples was approved by the Medical Ethics Committee of the Second Affiliated Hospital of Hainan Medical University (Ethical Licence number: LW2019312). Here, we solemnly declare that this study involved the detection of protein expression in human liver cancer samples, strictly in accordance with the Declaration of Helsinki. The research content and process of the project followed international and national ethical requirements for biomedical research.

### 2.2 Cell Lines and Animals

The human hepatoma cell line, Bel7402, which highly expresses AFP, was obtained from our laboratory. HLE cells that did not express AFP were obtained from the Kebai Biological Company (Nanjing, China). Cells were cultured in an incubator at 37°C and 5% CO_2_. Immunodeficient female BALB/c nude mice were purchased from Hunan Ansheng American Pharmaceutical Research Institute Co. Ltd. (Changsha, China). The mice were housed in the animal facility of Hainan Medical University. All animal experiments conducted in this study received ethical approval from the Experimental Animal Committee of Hainan Medical University. The animals involved in this study strictly comply with international and national biomedical research requirements and animal welfare.

### 2.3 Antibodies and Reagents

Sorafenib was purchased from Bayer Pharmaceutical Co. Ltd. (Germany). The following primary antibodies were used: anti‐AFP (Catalogue No. 14550‐1‐AP; Proteintech; 1:2000), anti‐PKM2 (15822‐1‐AP; Proteintech; 1:2000), anti‐HK2 (hexokinase 2, 22029‐1‐AP; Proteintech; 1:1000), anti‐GLUT1 (glucose transporter 1, Catalogue No. AWA00409; Aibivei Biotechnology Co. Ltd.; 1:1000), anti‐LDHA (lactate dehydrogenase A, Catalogue No. ER00702; Hangzhou Hua'an Biotechnology Co. Ltd.; 1:5000), and anti‐GAPDH (Catalogue No. 10094‐MM09; Sino Biological Inc.; 1:2000). Glucose consumption, lactate dehydrogenase (LDH) activity, and ATP generation measurement kits were purchased from Nanjing Jiancheng Biology Co. Ltd. (Nanjing, China).

### 2.4 Bioinformatics Analysis

The disparity in AFP expression between liver cancer tissues and adjacent tissues was examined using the timer online database (http://timer.cistrome.org/) and GEPIA online database (http://gepia.cancer‐pku.cn/).

### 2.5 Cell Transfection

Cells in the logarithmic growth phase were seeded in six‐well plates at a density of 3 × 10^6^ cells/well, and an appropriate amount of virus was added based on the multiplicity of infection (MOI) values determined in the pre‐experiment. After 72 h of lentiviral infection, puromycin was added to the first generation of the stably transfected cells.

### 2.6 Western Blotting

Total protein was extracted from the protein lysate at a temperature of 4°C. The protein concentration was determined using the bicinchoninic acid (BCA) method, and an appropriate amount of total protein was added and subjected to boiling at 100°C for 8 min. The extracted proteins were analyzed by sodium dodecyl sulfate‐polyacrylamide gel electrophoresis (SDS‐PAGE) and transferred onto polyvinylidene fluoride (PVDF) membranes. Subsequently, the membrane was incubated with the primary antibody overnight at 4°C and then with the secondary antibody for 2 h at room temperature. The membrane was developed using an electrogenerated chemiluminescence (ECL) luminescent solution.

### 2.7 MTT Colorimetry

Following overnight seeding of cells in the logarithmic growth phase in 96‐well plates at a concentration of 1 × 10^4^ cells/mL, sorafenib was added to the medium at concentrations of 2.5, 5, 10, 20, 30 and 40 μg/mL. The solute control and untreated wells were also included and incubated for 48 h. In a light‐avoidant environment, 20 μL of methyl thiazolyl tetrazolium (MTT) solvent was added to each well, followed by shaking with 150 μL dimethyl sulfoxide (DMSO) for 4 h for a duration of 10 min. The absorbance of each well was measured at a wavelength of 490 nm using a microplate reader. Based on the dose–response curves generated from this preliminary MTT assay and references from previously published literature, the working concentrations of sorafenib for subsequent functional experiments were determined as 3 μg/mL for HLE cells and 5 μg/mL for Bel7402 cells.

### 2.8 Cell Clone Formation

The cells (2 × 10^4^ cells/mL) were inoculated into 6‐well plates and cultured for 14 days. After 14 days, the cells were washed thrice with phosphate buffer saline (PBS) and fixed with 4% paraformaldehyde. After staining with crystal violet, the 6‐well plates were rinsed with water and the colonies were allowed to air dry before counting.

### 2.9 Scratch Experiment

Three parallel lines were drawn along the lower edge of the 6‐well plate with a marker pen separated by a distance of 1 cm. The cells were seeded at a density of 2 × 10^5^ cells/well. Following overnight incubation, the scratch on the aperture of the cell was aligned perpendicularly to the three horizontal lines located at the bottom. Subsequently, the medium was replaced every 24 h, and the cells were observed under an inverted microscope.

### 2.10 Transwell Assay

The lower chamber of a sterile 24‐well plate was filled with serum‐free culture medium and 5 × 10^4^ cells were added to the upper chamber. The cells were incubated for 48 h, fixed with 4% paraformaldehyde for 30 min, and stained with 0.1% crystal violet for 2 h. The cells in the upper chamber were removed using a cotton swab and photographed under an inverted microscope.

### 2.11 Flow Cytometry

After resuspension within the range of 5 × 10^4^ cells, the cells were counted and centrifuged at 1000 *g* for 5 min. The supernatant was discarded and 5 μL of PE Annexin V and 5 μL of 7‐AAD were added. Additionally, 400 μL of 1× binding buffer was resuspended. After incubation at room temperature for 20 min, the sample was placed in an ice bath and quickly analyzed using a computer.

### 2.12 The TUNEL Analysis

Cells (2 × 10^4^/mL) were inoculated into 24‐well plates and fixed with 4% paraformaldehyde after incubation for 48 h. Each well was treated with immunostaining force permeabilization solution for 5 min and then added to the prepared terminal deoxynucleotidyl transferase (TdT)‐mediated dUTP nick‐end labeling (TUNEL) assay solution, and incubated at 37°C in a 5% CO_2_ incubator for 60 min. At the end of the incubation period, 100 μL of 4′,6‐diamidino‐2‐phenylindole (DAPI) staining solution was used to stain the cells in the dark at room temperature for 30 min. After removing DAPI, 100 μL of anti‐fluorescence burst solution was added, and the cells were photographed under a fluorescence microscope.

### 2.13 Determination of Glucose Consumption, LDH Activity and ATP Production

After centrifugation, the supernatant was collected for assays. Glucose consumption, LDH activity, and ATP production were measured according to the manufacturer's instructions provided by the Nanjing Jiancheng Bioengineering Research Institute (Nanjing, China).

### 2.14 The Extracellular Acidification Rate (ECAR) Detection

The probe working solution reagent was prepared according to the instructions provided by Shanghai Beibo Biotechnology Company (Shanghai, China). The cultured cells were washed twice with assay buffer, and after discarding the assay buffer after the second wash, 90 μL of new assay buffer was used. The acidified fluorescent probe, P61 (10 μL), was added to each well and mixed thoroughly. The resulting cell plate was used to detect changes in extracellular acidification fluorescence.

### 2.15 Co‐Immunoprecipitation (Co‐IP)

After complete lysis of the cells, the supernatant was centrifuged for co‐immunoprecipitation (Co‐IP). Protein G magnetic beads were magnetically separated, drawn into the supernatant, and combined with an antibody working solution. The supernatant was collected, and after adding 500 μL TBS, it was separated on a magnetic frame for 10 s. The supernatant was extracted, and the lysate was subsequently introduced. For each 20 μL of magnetic beads, 100 μL of buffer was added and heated at 95°C for 5 min. Subsequently, the mixture was placed on a magnetic frame for 10 s to obtain the supernatant for immunoblotting.

### 2.16 Xenograft Tumour Model

A total of 1 × 10^7^ cells per group were suspended in 150 μL PBS and subsequently implanted subcutaneously into nude mice to establish a xenograft tumour model. Tumour length (*L*) and width (*W*) were measured at 3‐day intervals. After 28 days, 12 nude mice were euthanized and tumour nodules were extracted for further analysis. The length and width of the tumours were measured and their respective weights were recorded.

### 2.17 Immunohistochemistry (IHC)

The tissues were fixed in formalin, embedded in paraffin, and sectioned. Sections were incubated overnight at 4°C with the primary antibodies. Protein expression in HCC tissues was assessed using immunohistochemistry (IHC) detection and a semi‐quantitative scoring system. The scoring system included a classification based on the percentage of positive tumour cells, ranging from 0% to > 75%, and an intensity score ranging from negative to strong staining. Protein expression was evaluated independently by two experienced pathologists, who considered both staining intensity and scale scores in their comprehensive assessment.

### 2.18 Statistical Analysis

Statistical analyses were performed using SPSS (version 22.0; IBM Corp.). Quantitative data are expressed as the mean ± standard deviation (SD). Differences between two groups were assessed using an unpaired two‐tailed Student's *t*‐test. For comparisons involving three or more groups, one‐way analysis of variance (ANOVA) was applied, followed by Tukey's honestly significant difference (HSD) post hoc test for multiple comparisons. Graphs were generated using GraphPad Prism software (GraphPad Software Inc.). A *p*‐value of less than 0.05 (*p* < 0.05) was considered statistically significant.

## 3 Results

### 3.1 AFP Expression Is Significantly Upregulated in Human Liver Cancer Tissues and Is a Potential Biomarker of HCC

**FIGURE 1:**
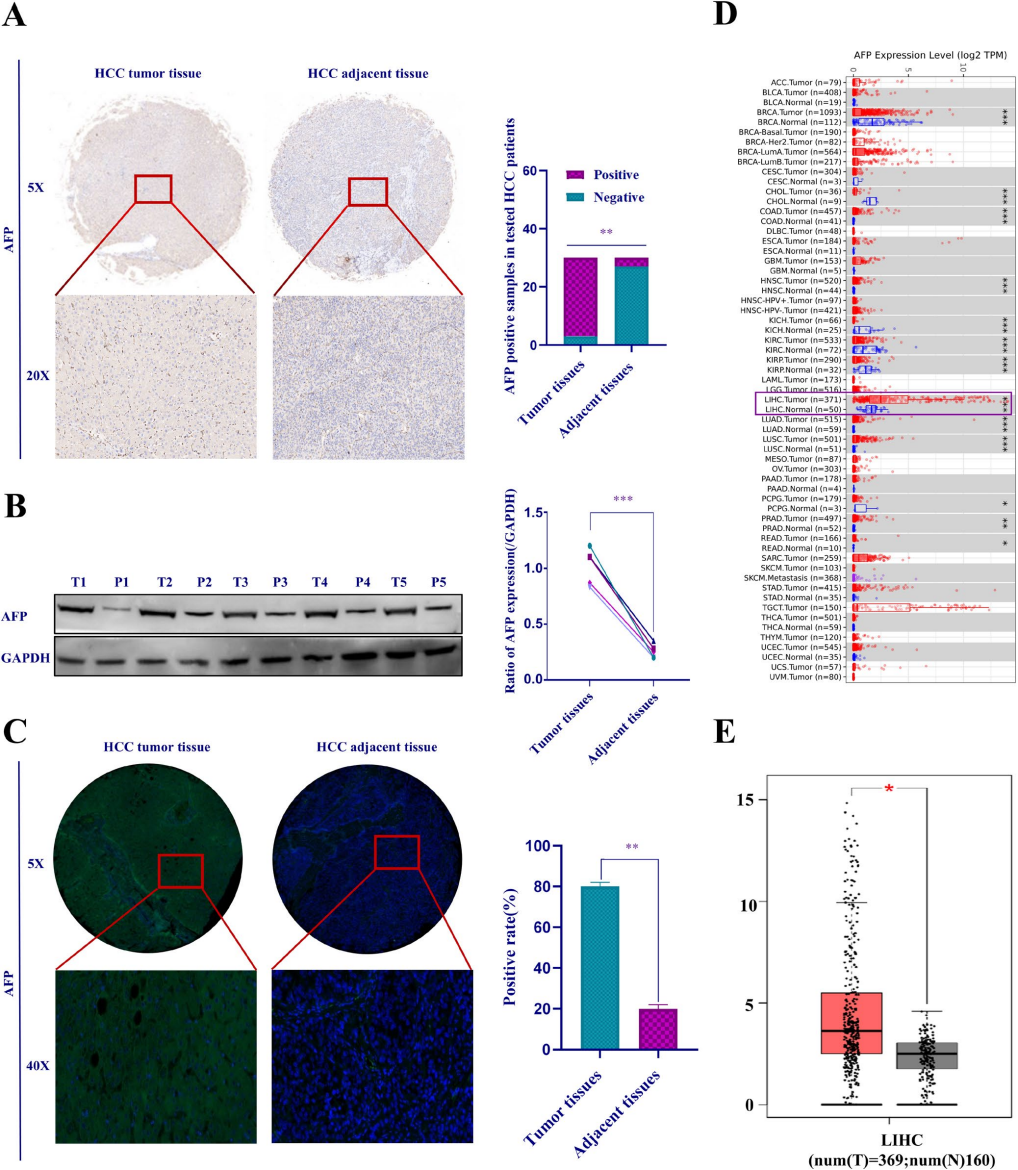
Expression of AFP in HCC tissues. (A) IHC staining to detect AFP expression in HCC tissues and corresponding adjacent (paracancerous) tissues, representative IHC images at different magnifications (left); statistical analysis of AFP positive rate in cancerous and adjacent tissues from HCC patients (right). (B) Western blotting analysis of AFP expression levels in 5 cases HCC tissues and adjacent tissues, with T representing tumour tissue and P representing adjacent tissue (left); statistics of AFP positive rate (right). (C) Immunofluorescence analysis showing nuclei in blue and AFP protein in green (left); statistical analysis of AFP positive rate (right). (D, E), mRNA expression of AFP in the timer and GEPIA databases, with red indicating tumour tissue and blue indicating adjacent tissue. **p < 0.01, ***p < 0.001.

To evaluate the expression level of AFP in human HCC tissues, we analyzed 30 pairs of HCC tissue microarray samples using immunohistochemistry (IHC), Western blotting, and immunofluorescence. The results indicated that AFP expression was significantly higher in HCC tissues than in adjacent (paracancerous) tissues (Figure 1A–C), which was further corroborated by the Tumour Immunology Assessment Resource (TIMER) database (Figure 1D) and the GEPIA database (Figure 1E). Consequently, AFP expression is significantly upregulated in human liver cancer tissues and is a potential biomarker of HCC.

### 3.2 AFP Mediated the Resistance of Sorafenib in HCC Cells

**FIGURE 2:**
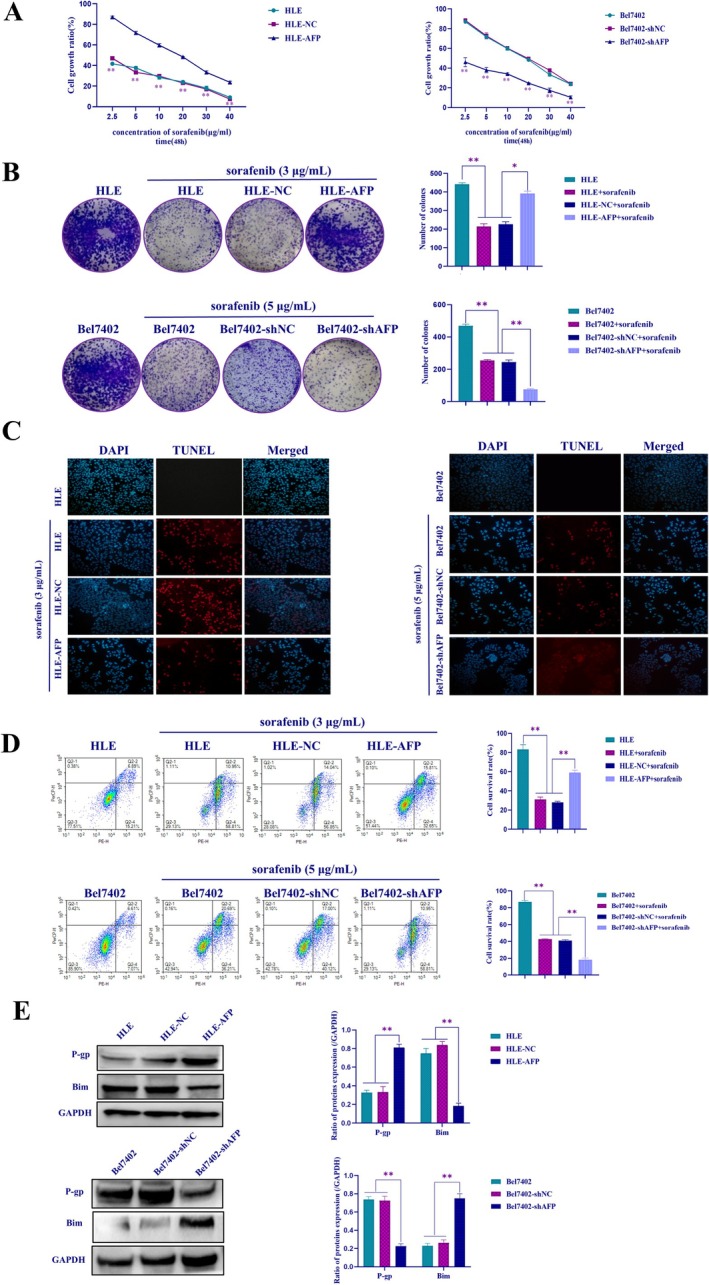
Effects of AFP on sorafenib inhibits the proliferation and promotes apoptosis of HCC cells. HLE, HLE‐NC and HLE‐AFP cells were treated with 3 μg/mL sorafenib for 48 h; while Bel7402, Bel7402‐shNC and Bel7402‐shAFP cells were treated with 5 μg/mL sorafenib for 48 h. (A) The cells' viability was assessed using the MTT assay; (B) the cells' proliferation was evaluated through cloning assay, the bar chart on the right shows statistical analysis; (C) TUNEL assay for detecting cell apoptosis of the HCC cells. (D) Flow cytometry for detecting the HCC cells' apoptosis rate, the bar chart on the right shows statistical analysis; (E) detection of changes in apoptosis‐related proteins by Western blotting assay, the bar chart on the right shows statistical analysis. *p < 0.05, **p < 0.01, ***p < 0.001. Data are presented as mean ± SD. The picture is a representation of three repeated experiments.

To explore the influence of AFP on the resistance of sorafenib in HCC cells, lentiviruses were employed to introduce AFP overexpression in HLE cells which lacked AFP expression, the result indicated that HLE cells higher expressed AFP while transfected with AFP‐expressed vectors than HLE cells transfected with negative control (NC) vectors in HLE cells (HLE‐NC), displayed HLE cell line with stable expression of AFP was successfully constructed (HLE‐AFP) (Figure S1A,B, left), whereas lentiviruses carrying the vectors that short hairpin RNA (shRNA) interfered with AFP expression (AFP‐shRNA) were used to infect Bel7402 cells which had high AFP expression, the result showed that AFP‐shRNA vectors were able to inhibit the expression of AFP in Bel7402 cells (Bel7402‐shAFP), but scramble sequence interfered vectors (shNC) could not inhibit the expression of AFP in Bel7402 cells (Bel7402‐shNC), it is suggested that the interference vectors which could inhibit AFP expression were successfully constructed (Figure S1A,B, right). According to the MTT (Figure 2A) and colony formation assays (Figure 2B), upregulation of AFP significantly enhanced the proliferation capacity of HCC cells and antagonized the cytotoxic effect of sorafenib, whereas interference with AFP significantly weakened the proliferation capacity and increased the cytotoxic effect of sorafenib in HCC cells. The scratch assay (Figure S2A) and transwell assay (Figure S2B) further confirmed that the overexpression of AFP promoted migration and antagonized the effect of sorafenib on the migration of HCC cells, whereas interfering with the expression of AFP could attenuate and synergise with sorafenib to restrain the migratory ability of HCC cells.

Furthermore, we investigated the effect of AFP on the apoptosis of HCC cells treated with sorafenib. TUNEL experiments (Figure 2C) and flow cytometry results (Figure 2D) showed that overexpression of AFP could antagonize sorafenib‐induced apoptosis of HCC cells. However, interfering with the expression of AFP could enhance sorafenib to promote the apoptosis of HCC cells. Western blotting was performed to analyze the influence of AFP on the expression of drug resistance‐related protein P‐gp and pro‐apoptosis related‐protein Bim while treated with sorafenib. The results indicated that overexpression of AFP was able to promote the expression of P‐gp and inhibited the expression of Bim in HLE cells; however, interference with AFP expression inhibited the expression of P‐gp and stimulated the expression of Bim in Bel7402 cells (Figure 2E). These results provided substantial evidence that AFP plays a pivotal role in sorafenib resistance in HCC cells.

### 3.3 AFP Stimulates Glucose Metabolic Reprogramming (GMR) in HCC Cells

**FIGURE 3:**
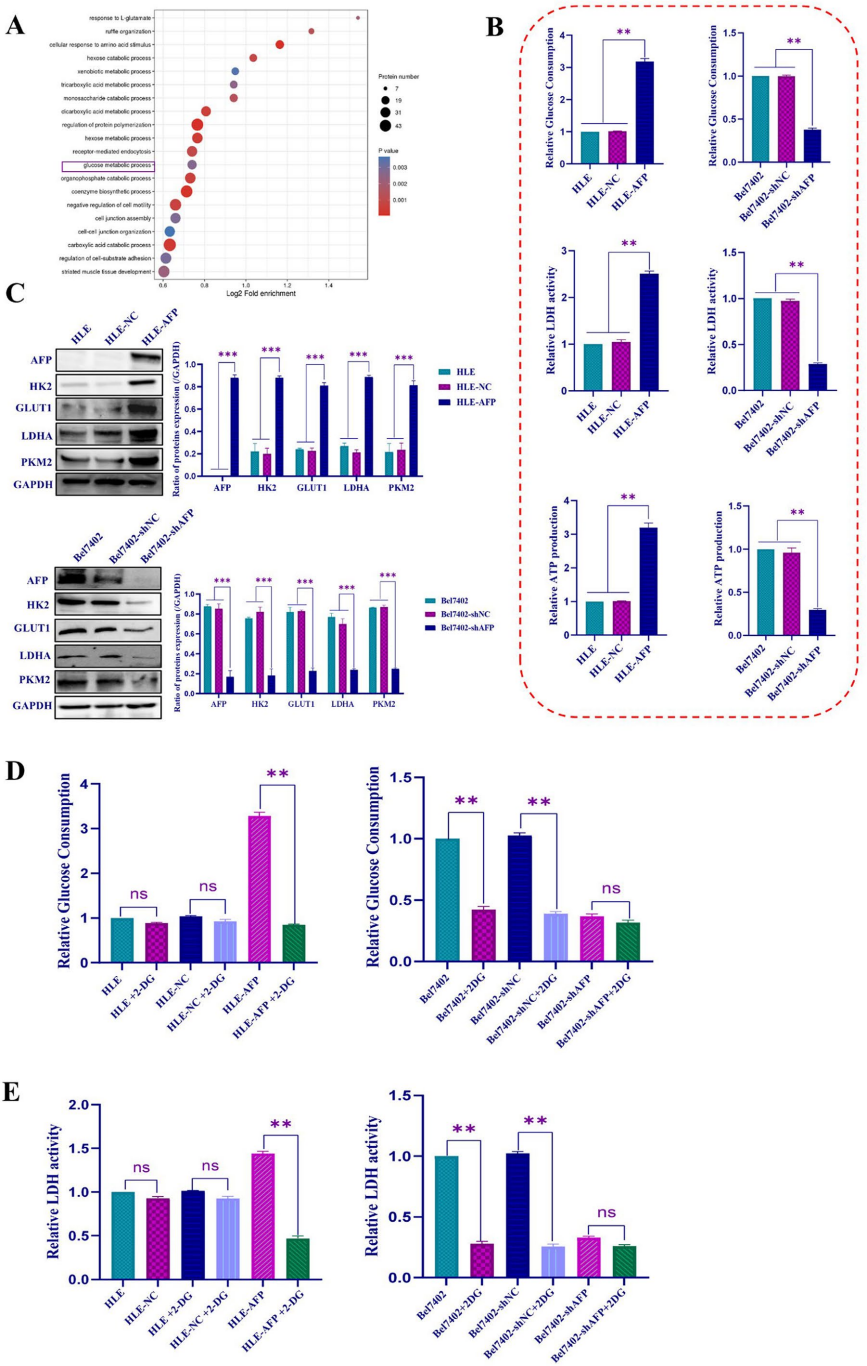
AFP regulates aerobic glycolysis in HCC cells. (A) Enrichment analysis results obtained from proteomic analysis of HLE‐NC cells and HLE‐AFP cells. The bubble plot depicts the KEGG pathway enrichment analysis of AFP and its co‐expressed genes, where the size of the dots represents the number of selected genes and the colour represents the *p*‐value of the enrichment analysis. (B) The glucose consumption, LDH activity and ATP production were detected in HLE, HLE‐NC and HLE‐AFP or Bel7402, Bel7402‐shNC and Bel7402‐shAFP cells, the changes in glucose consumption (upper), LDH activity (middle), and changes in ATP production (lower). (C) The expression changes of glycolysis‐related proteins were measured before and after overexpression of AFP in HLE cells and interfered the expression of AFP in Bel7402 cells. (D, E) The effect of 2‐DG on the glucose consumption and LDH activity in HLE, HLE‐NC and HLE‐AFP or Bel7402, Bel7402‐shNC and Bel7402‐shAFP cells were analysed by commercial test kit. Statistical plots of the effect of 2‐DG on glucose consumption (D) and LDH activity (E). **p < 0.01, ***p < 0.001. *N* = 3.

**FIGURE 4:**
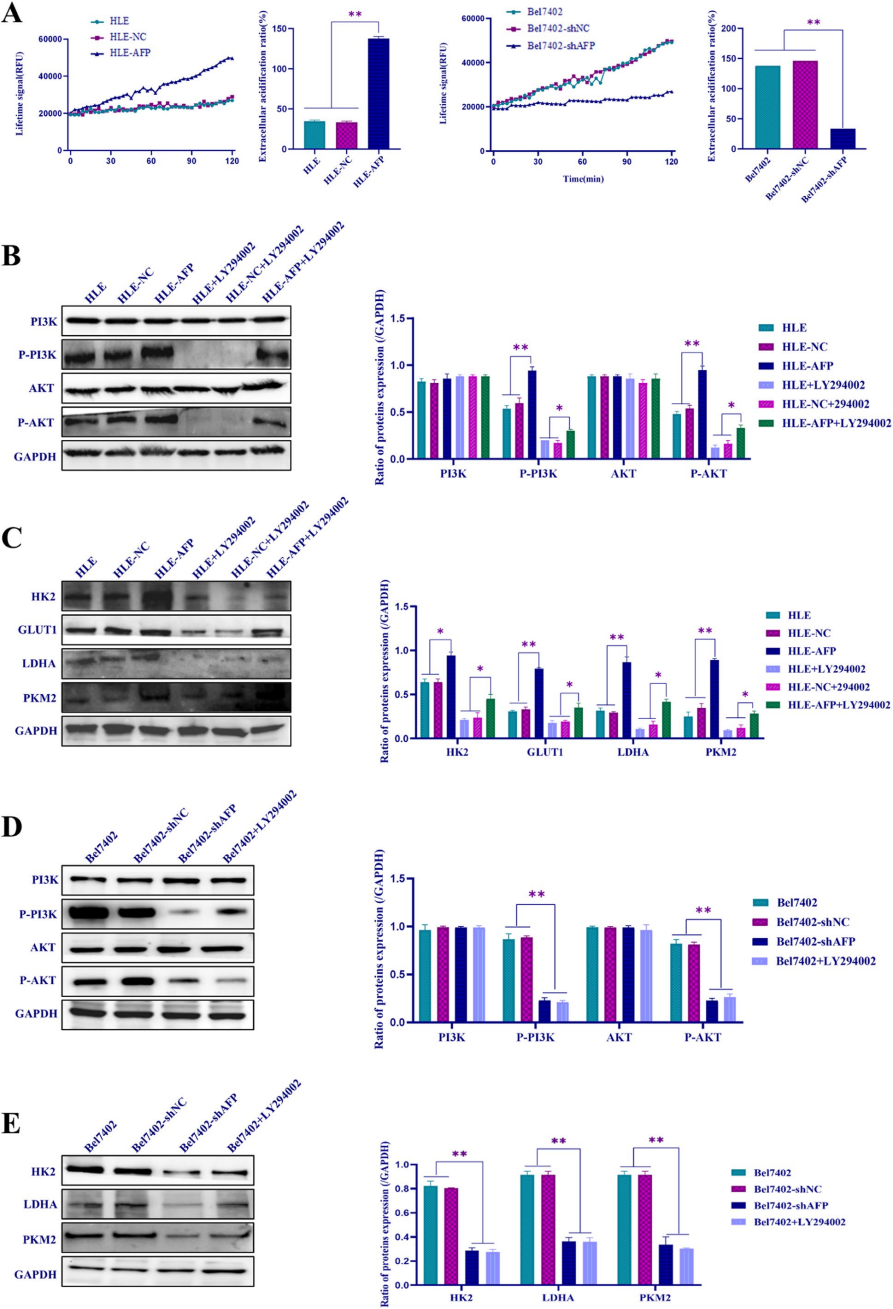
The changes of ECAR and the influence of AFP on the expression of GMR‐related proteins and the PI3K/AKT signalling pathway in HCC. (A) The extracellular acidification rate was determined by commercial test kit. Extracellular acidification rate (ECAR) curve analysis, difference in sample acidification rate before and after HLE cells overexpression of AFP (left) as well as before and after Bel7402 cells interfere with the expression of AFP (right). (B, C) Western blotting was used to detect the change of the PI3K/AKT signalling pathway and GMR‐related proteins expression after treated with the PI3K inhibitor, LY294002 for 12 h in HLE cells before and after overexpression of AFP, the bar chart on the right shows statistical analysis of protein grey scan value; (D, E) Western blotting was used to detect the change of the PI3K/AKT signalling pathway and GMR‐related proteins expression after treated with the PI3K inhibitor, LY294002 for 12 h in Bel7402 cells before and after interference with the expression of AFP, the bar chart on the right shows statistical analysis of protein grey scan value. *p < 0.05, **p < 0.01. The image represents the experiment repeated three times.

We analyzed the influence of AFP on the regulation of glucose metabolic reprogramming (GMR) in HCC cells. The results showed significant enrichment of AFP in genes associated with GMR (Figure 3A). Subsequently, measurements were taken for glucose consumption, lactate dehydrogenase (LDH) activity, and ATP production. Overexpression of AFP in HLE cells resulted in an elevated rate of glucose consumption, LDH activity, and ATP production (Figure 3B, left). Conversely, interference with AFP expression in Bel7402 cells led to a decrease in these parameters (Figure 3B, right), indicating that AFP can stimulate the GMR process. Subsequent Western blotting experiments demonstrated that the expression levels of GMR‐related proteins, namely HK2, GLUT1, LDHA and PKM2, increased in HLE cells following AFP overexpression (Figure 3C, upper panel). Conversely, in Bel7402 cells, interference with AFP expression led to a decrease in the expression of these proteins (Figure 3C, lower panel). Furthermore, glucose consumption analysis indicated that significantly increased glucose consumption in HLE cells when transfected with AFP‐expressing vectors, and glucose analog 2‐deoxy‐D‐glucose (2‐DG), which inhibits glycolysis, was applied to treat HLE‐AFP cells, showing that 2‐DG was able to suppress AFP stimulation of glucose consumption (Figure 3D, left). However, glucose consumption was significantly reduced in Bel7402 cells while silencing the expression of AFP (Figure 3D, right). LDH activity analysis showed that the activity of LDH was significantly elevated in HLE‐AFP cells, and 2‐DG inhibited this effect (Figure 3E, left) and interfered with the expression of AFP in Bel7402 cells, leading to a significant reduction in LDH activity (Figure 3E, right). The results also showed that 2‐DG blocked aerobic glycolysis in HCC cells, resulting in reduced glucose consumption and LDH activity across all groups (Figure 3D,E). Notably, this inhibitory effect of 2‐DG was more pronounced in HLE‐AFP cells and showed little change in Bel7402‐shAFP cells. These results further demonstrate that AFP can promote GMR in HCC cells. Additionally, in order to explore the influence of AFP on extracellular lactic acid production in HCC cells, we applied the extracellular acidification rate (ECAR) test to detect the concentration of lactic acid, and the results showed that AFP could significantly enhance ECAR in HCC cells (Figure 4A). Western blotting experiments indicated that the expression of phosphorylated PI3K (p‐PI3K), phosphorylated AKT (p‐AKT), HK2, GLUT1, LDHA and PKM2 were significantly elevated while HLE cells were transfected with AFP‐expressed vectors, and the PI3K inhibitor LY294002 could suppress the effect of AFP on the expression of these proteins (Figure 4B,C). However, the expression of these proteins was significantly decreased while interfering with the expression of AFP and treated with LY294002 in Bel7402 cells (Figure 4D,E). These results revealed that AFP stimulated activation of the PI3K/AKT signalling pathway to promote the expression of HK2, GLUT1, LDHA and PKM2. These findings suggested that AFP regulated the expression of GMR‐related proteins (enzymes) by activating the PI3K/AKT signalling pathway, ultimately promoting GMR in HCC cells.

### 3.4 AFP Promotes HCC Cells to Resist Sorafenib Through Stimulating Expression and Activity of PKM2

**FIGURE 5:**
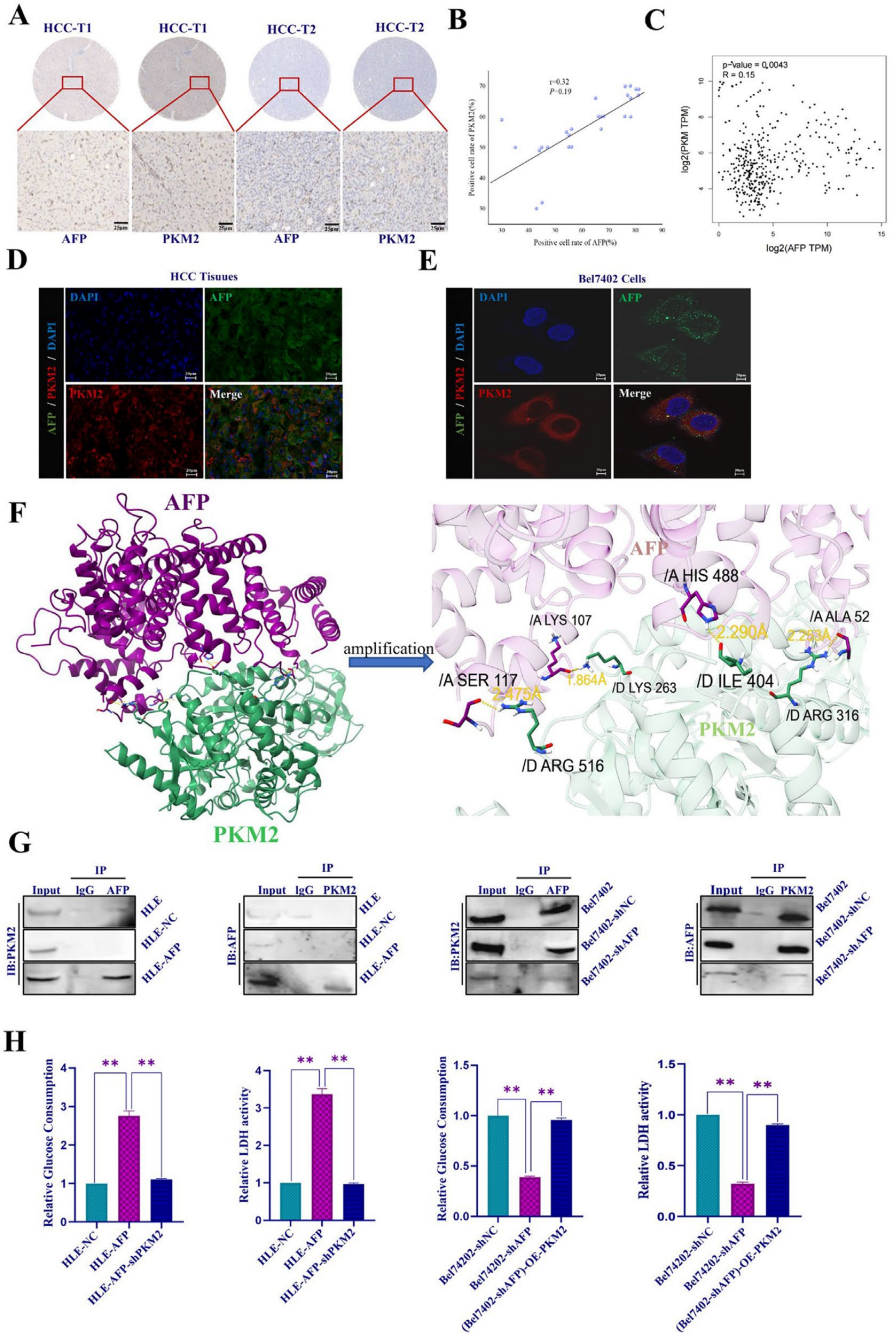
The interaction of AFP with PKM2, and the influence of PKM2 on AFP regulating GMR in HCC. (A) The expression of AFP and PKM2 was detected by immunohistochemistry in HCC tissues. (B) Two‐variable correlation analysis was performed in the SPSS 27.0 software; R positive value indicates a positive correlation between two variables. (C) Association analysis between AFP and PKM2 in the GEPIA database. (D, E) Immunofluorescence experiments were applied to observe the expression and co‐localization of AFP and PKM2 in HCC tissues and Bel7402 cells. F, Computer simulation and amino acid docking to analyse the interaction between AFP and PKM2. (G) The interaction of AFP and PKM2 was analysed by Co‐immunoprecipitation (Co‐IP). (H) HLE‐AFP cells were transfected with interfered PKM2 expressed vectors (HLE‐AFP‐shPKM2), and Bel7402‐shAFP cells were transfected with PKM2 overexpressed vectors ((Bel7402‐shAFP)‐OE‐PKM2); while the HCC cells were cultured for 48 h, the change of glucose consumption and LDH activity in these HCC cells were detected by commercial kits. **p < 0.01. *N* = 3.

The role of PKM2, a crucial enzyme regulating aerobic glycolysis, was examined in relation to sorafenib resistance in HCC cells induced by AFP. Initially, the association between AFP and PKM2 was investigated in HCC cells. A positive correlation between AFP and PKM2 expression was observed in 30 clinical histochemistry samples (Figure 5A,B) and the correlation analysis diagram of the GEPIA database (*p* = 0.0043, *R* = 0.15), indicating that the two variables AFP and PKM2 are correlated and weakly positively correlated (Figure 5C). In addition, immunofluorescence, immunohistochemistry analysis of liver cancer tissues (Figure 5D) and Bel7402 cells (Figure 5E), the results showed that high expression of AFP and PKM2, and the proteins were co‐localized in the cytoplasm of liver cancer tissues and HCC cell lines. To analyze the effect of AFP on the activity of PKM2, we performed computer simulation and amino acid docking analysis. The results indicated that there is an amino acid interaction between AFP and PKM2. These results suggest that the interaction between AFP and PKM2 may affect the enzymatic activity of PKM2 (Figure 5F). Further, we transfected AFP‐expressing vectors into HLE cells, and then interfered with PKM2 expression, which was interfered with in Bel7402 cells and transfected into PKM2 overexpression vectors (OE‐PKM2), indicating that shPKM2 vectors were successfully transfected into HLE‐AFP (HLE‐AFP‐shPKM2) cells (Figure S3A, left) and OE‐PKM2 into Bel7402‐shAFP ((Bel7402‐shAFP)‐OE‐PKM2) cells (Figure S3A, right). Western blotting analysis showed that the expression of PKM2 was significantly lower in HLE‐AFP‐shPKM2 than in HLE‐AFP cells (Figure S3B, left), and the expression of PKM2 was significantly higher in (Bel7402‐shAFP)‐OE‐PKM2 than in Bel7402‐shAFP cells (Figure S3B, right). Co‐immunoprecipitation (Co‐IP) was performed to analyze the interaction between AFP and PKM2. The results also confirmed the interaction between AFP and PKM2 in HLE and Bel7402 cells (Figure 5G). In the present study, we determined whether PKM2 could enhance the influence of AFP on GMR in HCC cell lines with stable knockdown of PKM2 expression in HLE‐AFP cells, and whether HCC cell lines were established with stable overexpression of PKM2 in Bel7402‐shAFP cells. The results indicated a significant decrease in glucose consumption and LDH activity in HLE‐AFP cells following PKM2 knockdown (HLE‐AFP‐shPKM2) (Figure 5H, left), whereas Bel7402‐shAFP cells overexpressing PKM2 ((Bel7402‐shAFP)‐OE‐PKM2) exhibited overexpression of PKM2, which effectively counteracted the decrease in glucose consumption and LDH activity caused by the inhibition of AFP expression (Figure 5H, right). These results displayed that AFP was able to promote glucose consumption and LDH activity in HCC cells.

**FIGURE 6:**
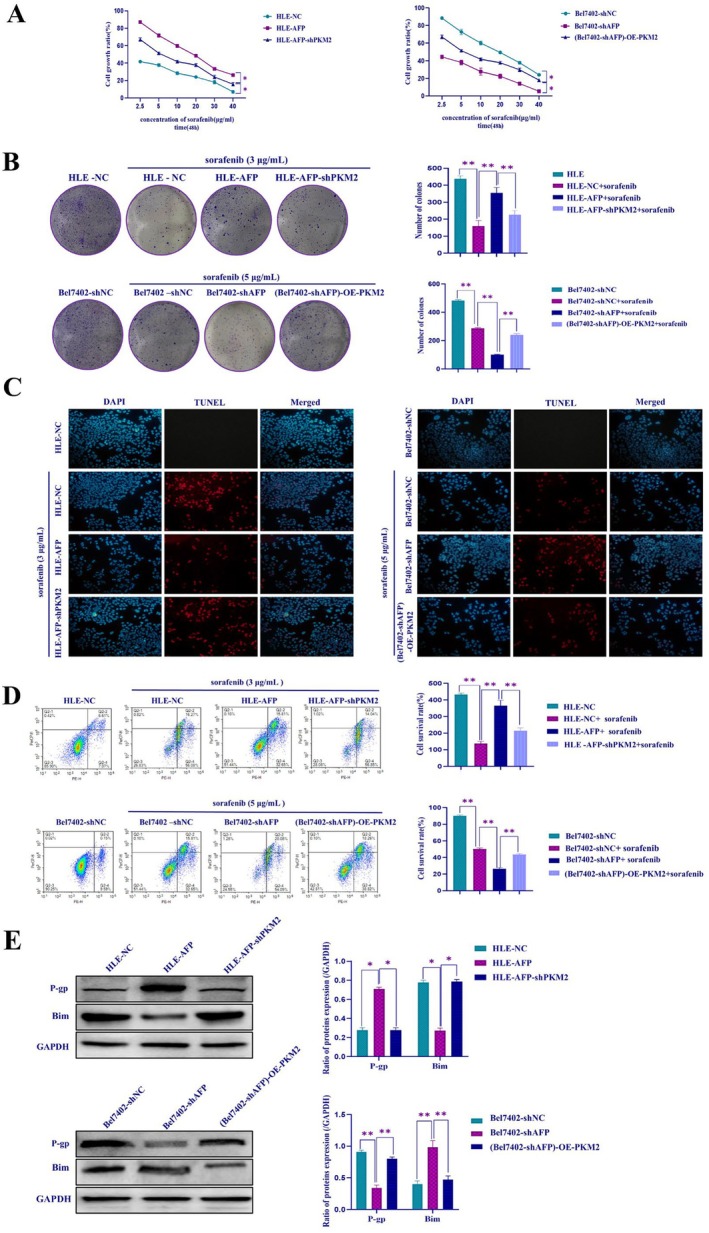
The influence of PKM2 on AFP‐mediated sorafenib resistance in HCC cells. (A) HLE, HLE‐AFP and HLE‐AFP‐shPKM2 cells, or Bel7402, Bel7402‐shAFP and (Bel7402‐shAFP)‐OE‐PKM2 were treated with different concentrations of sorafenib for 48 h. (A) The cells' viability was assessed using the MTT assay; (B) cellular colony formation assay was used to detect cell colony formation ability, the bar chart on the right shows statistical analysis; (C) the apoptosis of HCC cells was measured by TUNEL method; (D) the change in the cell apoptosis rate of HCC cells was determined by flow cytometry, the bar chart on the right shows statistical analysis. (E) The expression change of drug resistance related protein P‐gp and pro‐apoptotic related protein Bim were determined by Western blotting, the bar chart on the right shows statistical analysis. *p < 0.05, **p < 0.01. The image represents the experiment repeated three times.

Subsequently, we evaluated the effect of AFP and PKM2 on sorafenib‐mediated inhibition of HCC cell proliferation and migration. The results of the MTT assay (Figure 6A), plate cloning assay (Figure 6B), scratch assay (Figure S4A), and transwell assay (Figure S4B) showed that the disruption of PKM2 effectively counteracted the pro‐proliferative and pro‐invasive effects induced by AFP and enhanced the sensitivity of HCC cells to sorafenib. In contrast, overexpression of PKM2 could effectively reverse the inhibition of cell proliferation and invasion due to interference with AFP expression and reduce the sensitivity of HCC cells to sorafenib. These results implied that the expression of AFP in HCC cells may inhibit the proliferation and migration of HCC cells, and overexpression of PKM2 has the same effect.

To further investigate the role of PKM2 in the regulation of sorafenib resistance in HCC cells, in the present study, we applied a TUNEL assay (Figure 6C) and flow cytometry (Figure 6D) to assess cell survival and apoptosis following treatment with sorafenib. The results showed that in the drug‐treated group, HLE‐AFP cells exhibited the highest survival rate and the lowest apoptosis rate compared to HLE‐NC cells. However, HLE‐AFP‐shPKM2 cells exhibited lower survival and higher apoptosis rates than those of HLE‐AFP cells. Similarly, Bel7402‐shAFP cells exhibited lower survival and higher apoptosis rates than Bel7402‐shNC cells. However, in Bel7402‐shAFP cells, overexpression of PKM2 (Bel7402‐shAFP‐OE‐PKM2) resulted in a significantly higher survival rate and lower apoptosis rate compared to Bel7402‐shAFP. Western blotting analysis revealed that overexpression of AFP in HLE cells resulted in a partial increase in P‐gp expression and a decrease in Bim expression, which was partially reversed by the expression of PKM2. Conversely, overexpression of PKM2 in Bel7402‐shAFP cells led to a partial increase in the expression of P‐gp and a decrease in the expression of Bim in HCC cells (Figure 6E). These findings demonstrated that interfering with PKM2 is capable of counteracting the facilitative effect of AFP on sorafenib resistance in HCC cells, thereby implying that AFP might induce drug resistance by stimulating the GMR via activating PKM2 in HCC cells.

### 3.5 Inhibited Expression of AFP Was Able to Reduce Tumorigenesis and Growth of HCC, Which Was Restored by Overexpression of PKM2 In Vivo

**FIGURE 7:**
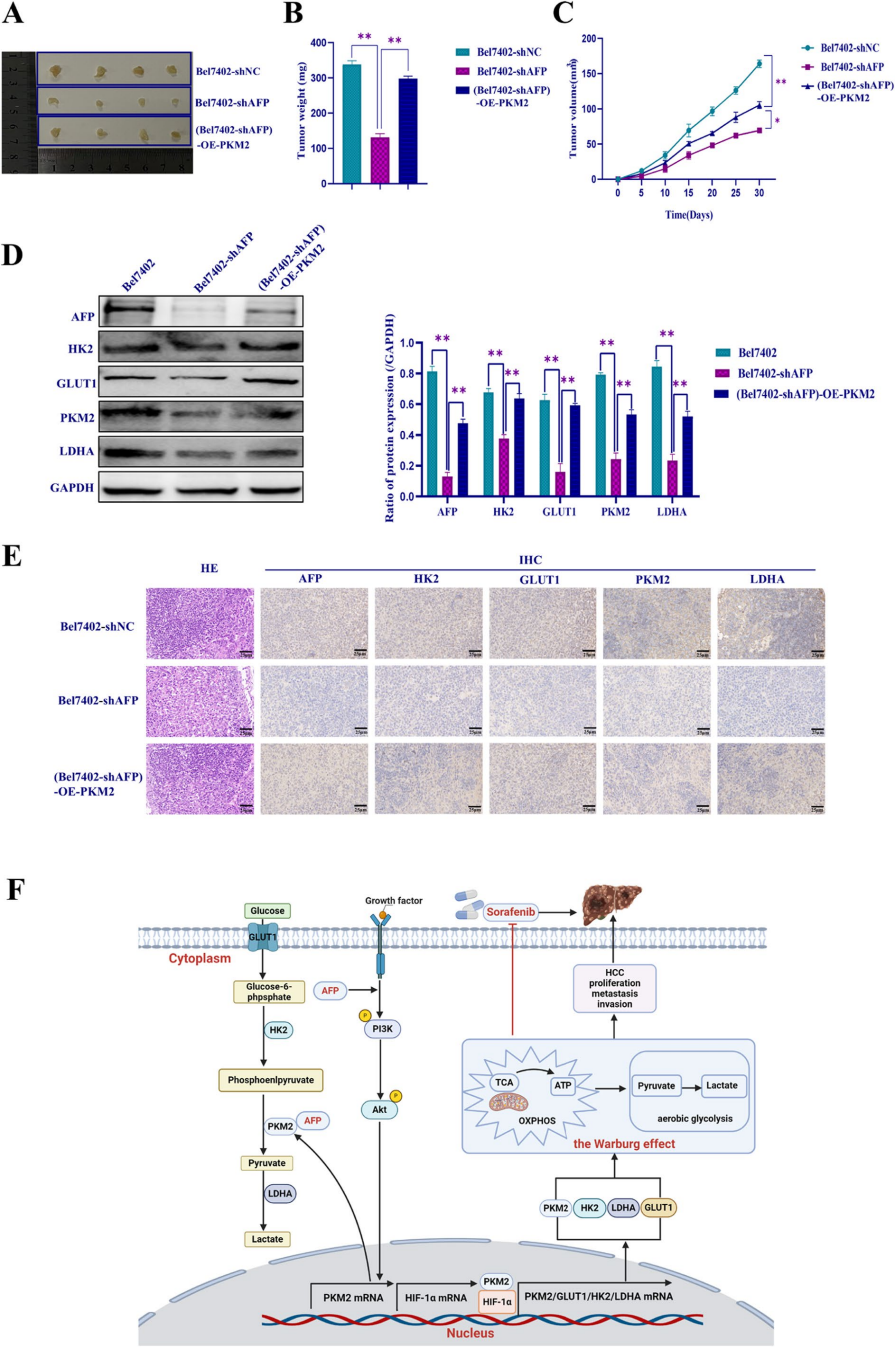
The influence AFP and PKM2 on mouse subcutaneous tumour model and the expression of GMR‐related proteins in vivo. (A) Subcutaneous injection of HCC cells to establish xenograft tumour models in nude mice. (B) Bar chart showing the statistical analysis to evaluate tumour weights. (C) Growth curve of the tumour volume in subcutaneous tumour formation for statistical analysis. (D) Proteins extracted from xenografts tumour tissues of nude mice were subjected to Western blotting analysis to detect the expression change of GMR‐related proteins (HK2, GLUT1, PKM2 and LDHA). (E) Immunohistochemistry experiments examined GMR‐related proteins expression in mouse tumour tissues. *p < 0.05, **p < 0.01. (F) Schematic illustrating the role mechanism of AFP in promoting GMR contributing to HCC cells' resistance to sorafenib. AFP activates the PI3K/AKT signalling pathway, promotes the expression of GMR‐related proteins (PKM2, HK2, GLUT1, LDHA), thereby stimulating the progression of GMR and enhancing the resistance of HCC cells to sorafenib.

To investigate the effects of PKM2 overexpression on tumorigenesis and growth after AFP knockdown in vivo, we established a nude‐mouse xenograft model. We injected three control cell lines, Bel7402‐shNC, Bel7402‐shAFP, and (Bel7402‐shAFP)‐OE‐PKM2 (Figure 7A). The results showed that PKM2 overexpression restored the decrease in tumour volume and weight caused by interfering with AFP expression (Figure 7B,C). Next, we conducted Western blotting and immunohistochemistry experiments on mouse tumour tissues to detect the expression levels of GMR‐related proteins. The results indicated that the overexpression of PKM2 restored the downregulation of GMR‐related proteins caused by the suppression of AFP in vivo (Figure 7D,E). Therefore, these findings suggested that PKM2 overexpression could restore the reduced ability of tumorigenesis and growth in vivo in the absence of AFP.

## 4 Discussion

HCC is regarded as a highly malignant tumour [34]. The malignant behaviours of HCC have six characteristics: sustained proliferation, escape from growth inhibition, immortality, angiogenesis, invasion, metastasis, and the Warburg effect [35, 36]. The enhancing effect of aerobic glycolysis in HCC has been widely reported, as aerobic glycolysis can provide a more effective energy and metabolic substrate supply for tumour proliferation, and the production of lactic acid leads to an acidic environment that promotes metastasis and evades immune surveillance of cancer cells [37, 38]. Therefore, changes in cellular metabolism have become hallmarks of cancer.

AFP, a member of the HSA family, is an important tumour marker for primary HCC with multiple biological functions including the promotion of cell proliferation, metastasis and immune escape. When activated, AFP acts as a signalling molecule to trigger other physiological processes and cellular events. Although some researchers have found that AFP is closely associated with poor prognosis in patients with HCC [39, 40, 41], the specific role of AFP in the progression of HCC is unknown. Several studies have found that AFP is closely related to multidrug resistance [42, 43, 44]. Considering the resistance of HCC cells to sorafenib, we hypothesized that AFP is a key factor in the development of sorafenib resistance in patients with HCC. We confirmed the expression of AFP in HCC tumour tissues at the clinical and cellular levels and found that AFP affects the resistance of HCC cells to sorafenib.

Cancer cells exhibit an enhanced glycolytic phenotype during tumour progression [45]. Inhibition of the Warburg effect can greatly suppress tumorigenicity and promote cancer cell apoptosis [46]. We hypothesized that AFP is a key molecule that regulates glycolytic processes and promotes sorafenib resistance by stimulating the Warburg effect. First, we explored the mechanism by constructing HCC cells overexpressing and interfering with the expression of AFP and observed the expression of GLUT1 and the activity of LDH as well as proteins related to the Warburg effect in HCC cells. This study's results showed that AFP increased aerobic glycolysis by promoting glucose consumption, LDH activity, and ATP production in HCC cells and also promoted the expression of GMR‐related proteins. These results suggested that AFP promotes HCC cell aerobic glycolysis.

To further explore the regulatory effect of AFP on GMR in HCC cells, we used the glycolytic blocker 2‐DG to inhibit glycolysis. These results showed that AFP could stimulate the production of ATP by inducing aerobic glycolysis in HCC cells. During glycolysis, pyruvate is converted to lactate, resulting in the net production of protons in the extracellular medium, forming an acidic medium [47]. Therefore, we used an ECAR assay to measure the rate of extracellular acidification and to determine the rate of glycolysis and GMR capacity of HCC cells. The results displayed that AFP increased glycolysis in HCC cells. Therefore, we concluded that targeting AFP could reduce glycolysis and the acidification rate of HCC, restraining cancer cells' proliferation and metastasis.

The molecular mechanism regulating the GMR in cancer cells is complex. Studies have shown that the PI3K/AKT signalling pathway may regulate glucose metabolism and promote glucose uptake in tumour cells [48, 49]. Previously, we have found that AFP could activate the PI3K/AKT signalling pathway, promoting the occurrence and development of liver cancer [50]. Therefore, we investigated whether AFP was able to activate the PI3K/AKT signalling pathway to stimulate the expression of GMR‐related proteins. The results showed that AFP may promote cellular aerobic glycolysis by activating the PI3K/AKT signalling pathway. GMR is an extremely complex process that may involve multiple molecular mechanisms; in this study, HCC cells were selected for the experiments, and a combination of glycolysis blocker 2‐DG and the PI3K/AKT signalling pathway inhibitor (LY29002) was used to investigate the regulatory effect of AFP on GMR in HCC cells. The results showed that 2‐DG and LY29002 were able to inhibit AFP‐stimulated aerobic glycolysis in HCC cells, implying that the PI3K/AKT signalling pathway is an important factor in promoting GMR in HCC cells. These results indicated that the regulated mechanism of GMR in HCC may involve the activation of the PI3K/AKT signalling pathway.

The activation of the Warburg effect is an important characteristic of cancer cells. PKM2 is a key enzyme for regulating the Warburg effect; evidence has shown that the overexpression of PKM2 has been widely recognized in HCC and is associated with the resistance to sorafenib [51, 52]. We further investigated whether AFP regulates PKM2 expression, alters glucose metabolism, and exerts resistance to sorafenib. We found a positive correlation between AFP and PKM2 expression at the clinical tissue and in the GEPIA database. Computer simulation and amino acid docking analysis, Co‐IP, and immunofluorescence experiments confirmed that there was an amino acid interaction between AFP and PKM2, and co‐localization of AFP and PKM2 in the cytoplasm, implying that AFP may stimulate the activity of PKM2 to promote GMR in HCC cells. Although AFP is classically recognized as a secreted glycoprotein, emerging evidence reveals that a substantial fraction of unsecreted AFP is retained in the cytoplasm, where it functions as a critical intracellular signalling molecule. Previous studies have demonstrated that this cytoplasmic pool of AFP can directly interact with intracellular proteins, such as PTEN and retinoic acid receptors, to modulate cellular survival and signalling cascades [17, 32]. Consistent with these findings, our spatial observations suggest that unsecreted cytoplasmic AFP interacts directly with the cytosolic enzyme PKM2, providing a novel subcellular basis for AFP‐mediated metabolic reprogramming. In addition, we found that AFP promoted the Warburg effect by inducing the formation of PKM2 dimers, and analyzed the role of PKM2 in the Warburg effect by detecting glucose consumption and LDHA activity. The results showed that the overexpression of PKM2 could reverse the effect of the attenuation in the proliferation and migration ability of HCC cells caused by interference with AFP expression or sorafenib treatment; the opposite effect was observed for interference with the expression of PKM2. In addition, in vivo experiments further verified that PKM2 overexpression restores the development and growth of xenograft tumours in mice. These results further confirmed that AFP and PKM2 may act synergistically to promote sorafenib resistance in HCC. It should be noted that the clinical validation in this study was based on a relatively small cohort of 30 HCC tissue pairs. Future studies with larger, multi‐center clinical cohorts are warranted to further validate the clinical significance of the AFP‐PKM2 axis. Inhibition of PKM2 activity is a key intracellular factor in suppressing AFP‐stimulated malignant behaviours of HCC cells by promoting GMR. The molecular mechanism by which AFP stimulates GMR leads to sorafenib resistance in HCC, as shown in Figure 7F. This study is the first time to report that AFP interacts with PKM2, and AFP is able to stimulate the activity of PKM2 to promote GMR, contributing to HCC resistance to sorafenib.

## 5 Conclusions

This study found that AFP was a key factor for promoting the resistance of sorafenib in HCC. The results also identified that AFP was a key molecule for regulating GMR, and that the AFP‐PKM2 axis plays an important role in the resistance of sorafenib in HCC. This study reveals the molecular mechanism by which AFP promotes the expression of PKM2 by activating the PI3K/AKT signalling pathway, stimulates PKM2 activity, accelerates the progression of GMR, and contributes to the tolerance of HCC cells to sorafenib. PKM2 was identified as an important downstream target that interacts with AFP. This study provides a theoretical breakthrough in the field of overcoming drug resistance in liver cancer and provides a new perspective for elucidating the GMR regulation mechanism of HCC, targeting AFP for the precise clinical treatment of liver cancer.

## Author Contributions

**Yuli Zhou:** investigation, writing – original draft, methodology, data curation. **Yi Chen:** conceptualization, investigation, data curation. **Wei Li:** investigation, data curation. **Qiushi Yin:** conceptualization, investigation, resources, data curation. **Xu Dong:** data curation, investigation. **Mengsen Li:** conceptualization, writing – review and editing, supervision, funding acquisition, project administration. **Mingyue Zhu:** conceptualization, funding acquisition, writing – review and editing, supervision, data curation. **Kun Liu:** data curation, writing – original draft, funding acquisition. **Siren Feng:** investigation, writing – original draft, data curation. **Bo Lin:** conceptualization, investigation, funding acquisition, formal analysis.

## Funding

This project was supported by the National Natural Science Foundation of China (Nos. 82460602, 82573045, 82560459 and 82060514); the Natural Science Foundation of Hainan Province (No. 824RC517); Hainan Provincial Science and Technology Special Fund (No. ZDYF2021SHFZ222); Joint Project of Hainan Provincial Health Science and Technology (No. WSJK2025MS132) and Hainan Provincial Graduate Innovation Project (Qhyb2023‐175).

## Conflicts of Interest

The authors declare no conflicts of interest.

## Supporting information


**Figure S1:** The effect of interference or overexpression vectors on the expression of AFP in HCC cells. (A) HLE cells were transfected with negative control (NC) vectors (HLE‐NC), and HLE cells transfected with AFP‐expressed vectors (HLE‐AFP); while Bel7402 cells were transfected with scramble sequence vectors for interfering the expression of AFP (Bel7402‐shNC) and Bel7402 cells were transfected with AFP interfered sequence vectors (Bel7402‐shAFP), fluorescence observation of HLE cells was examined both before and after AFP overexpression, as well as in Bel7402 cells before and after AFP silencing. (B) The transfection effect of the cells was confirmed through Western blotting experiments, the bar chart on the right shows statistical analysis of protein grey scan value. **p < 0.01, ***p < 0.001. The picture represents the results of three repeated experiments.

**Figure S2:** The effects of AFP on sorafenib inhibits the migration of HCC cells. HLE, HLE‐NC, and HLE‐AFP cells were treated with 3 μg/mL sorafenib, whereas Bel7402 and Bel7402‐shNC and Bel7402‐shAFP cells were treated with 5 μg/mL sorafenib for 48 h. (A) Scratch assay to detected the damage repair of HCC cells, the bar chart on the right shows statistical analysis. (B) Transwell assay were used to determine the migratory ability of HCC cells after treated with sorafenib, the bar chart on the right shows statistical analysis. *p < 0.05, **p < 0.01, ***p < 0.001. The image represents the experiment repeated three times.

**Figure S3:** The effect of interference or overexpression vectors on the expression of PKM2 in HCC cells. (A) Transfection efficiency in HLE‐AFP cells or Bel7402‐shAFP cells were observed using a fluorescence microscope. (B) The efficiency of PKM2 interference in HLE‐AFP cells or overexpression in Bel7402‐shAFP cells were verified by Western blotting, the bar chart on the right shows statistical analysis of protein grey scan value. **p < 0.01. The image represents the experiment repeated three times.

**Figure S4:** The effect of PKM2 on AFP antagonizing the inhibitory effect of sorafenib on the migration of HCC cells. HLE, HLE‐AFP and HLE‐AFP‐shPKM2 cells were treated with sorafenib (3 μg/mL), or Bel7402, Bel7402‐shAFP and (Bel7402‐shAFP)‐OE‐PKM2 cells were treated with sorafenib (5 μg/mL) for 48 h, and the scratch test (A) and transwell test (B) are used to detect the migratory ability of HCC cells, the bar chart on the right shows statistical analysis. *p < 0.05, **p < 0.01. The picture represents the results of three repeated experiment.

## Data Availability

The datasets supporting the findings of this study are available in the TIMER (http://timer.cistrome.org/) and GEPIA (http://gepia.cancer‐pku.cn/) databases.
