# Supplementary figures and images for: AFP Stimulates Glucose Metabolic Reprogramming Contributing to Hepatocellular Carcinoma Resist Sorafenib Through Activating PI3K/AKT Signalling Pathway
Source: J Cell Mol Med. 2026 Jun 4;30(11):e71226. doi: 10.1111/jcmm.71226 (PMC13238858; doi:10.1111/jcmm.71226)

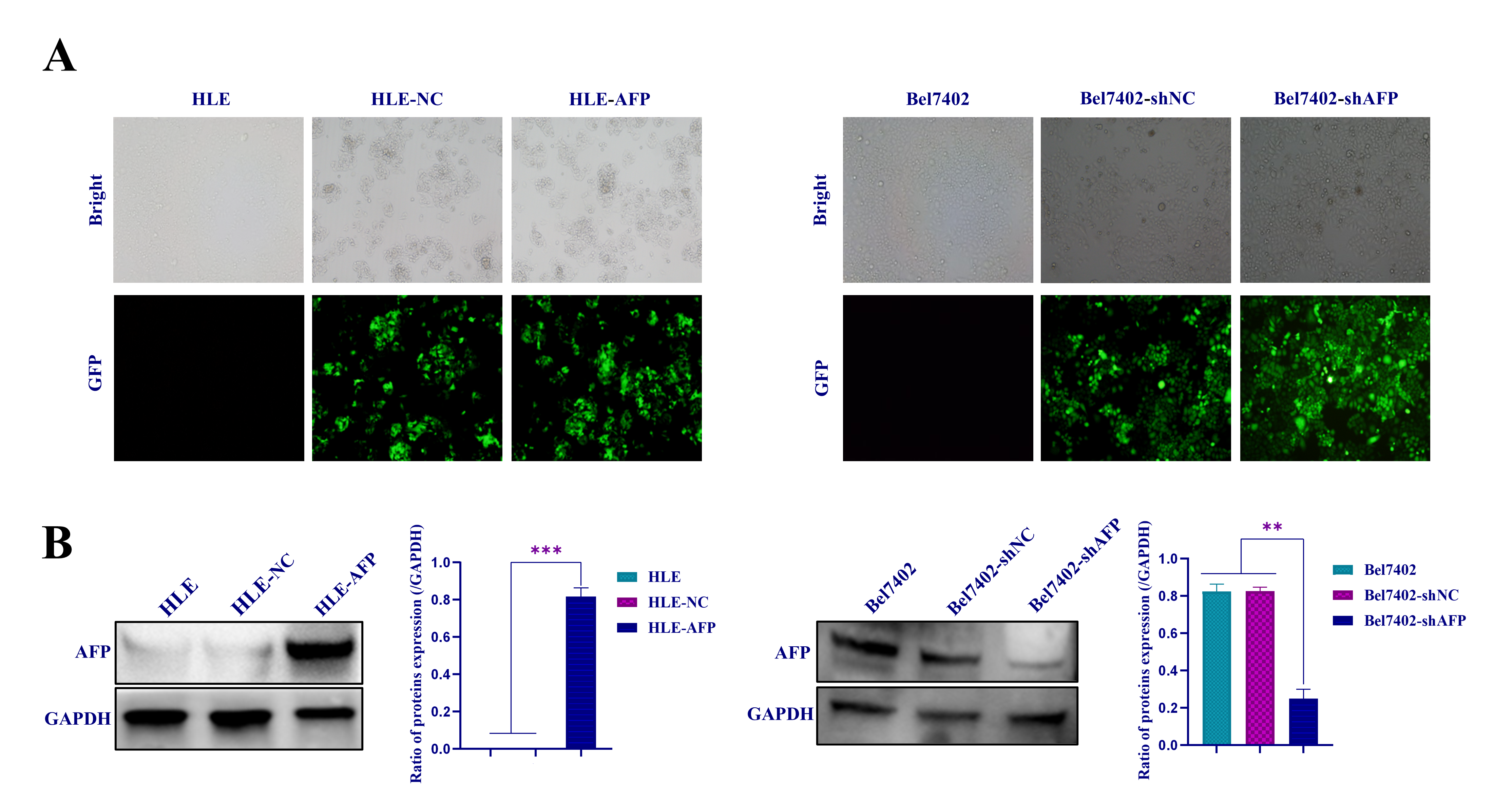

Supplement: Supplementary file 1 — Figure S1: The effect of interference or overexpression vectors on the expression of AFP in HCC cells. (A) HLE cells were transfected with negative control (NC) vectors (HLE‐NC), and HLE cells transfected with AFP‐expressed vectors (HLE‐AFP); while Bel7402 cells were transfected with scramble sequence vectors for interfering the expression of AFP (Bel7402‐shNC) and Bel7402 cells were transfected with AFP interfered sequence vectors (Bel7402‐shAFP), fluorescence observation of HLE cells was examined both before and after AFP overexpression, as well as in Bel7402 cells before and after AFP silencing. (B) The transfection effect of the cells was confirmed through Western blotting experiments, the bar chart on the right shows statistical analysis of protein grey scan value. **p < 0.01, ***p < 0.001. The picture represents the results of three repeated experiments. [file JCMM-30-e71226-s002.tif]

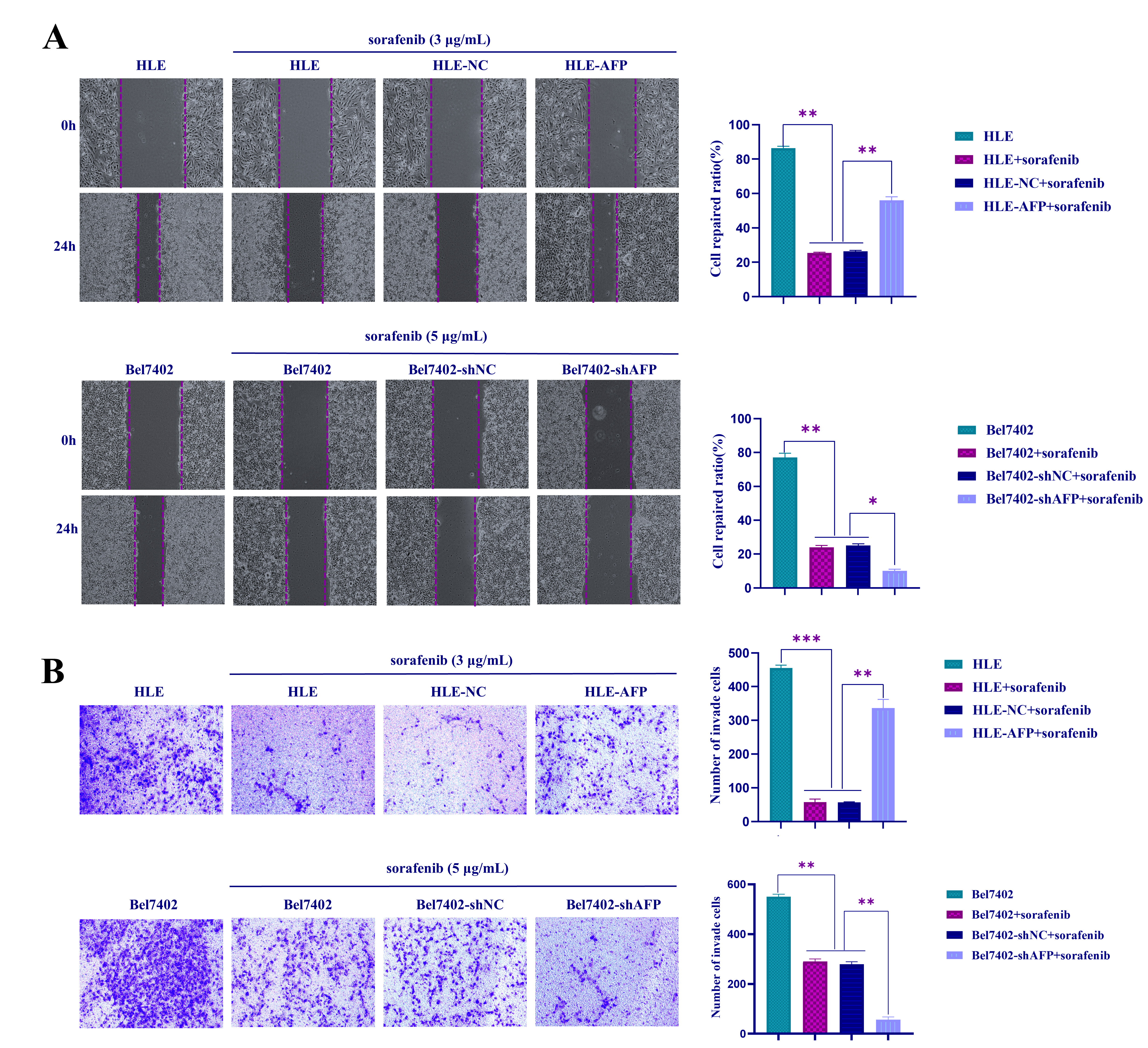

Supplement: Supplementary file 2 — Figure S2: The effects of AFP on sorafenib inhibits the migration of HCC cells. HLE, HLE‐NC, and HLE‐AFP cells were treated with 3 μg/mL sorafenib, whereas Bel7402 and Bel7402‐shNC and Bel7402‐shAFP cells were treated with 5 μg/mL sorafenib for 48 h. (A) Scratch assay to detected the damage repair of HCC cells, the bar chart on the right shows statistical analysis. (B) Transwell assay were used to determine the migratory ability of HCC cells after treated with sorafenib, the bar chart on the right shows statistical analysis. *p < 0.05, **p < 0.01, ***p < 0.001. The image represents the experiment repeated three times. [file JCMM-30-e71226-s003.jpg]

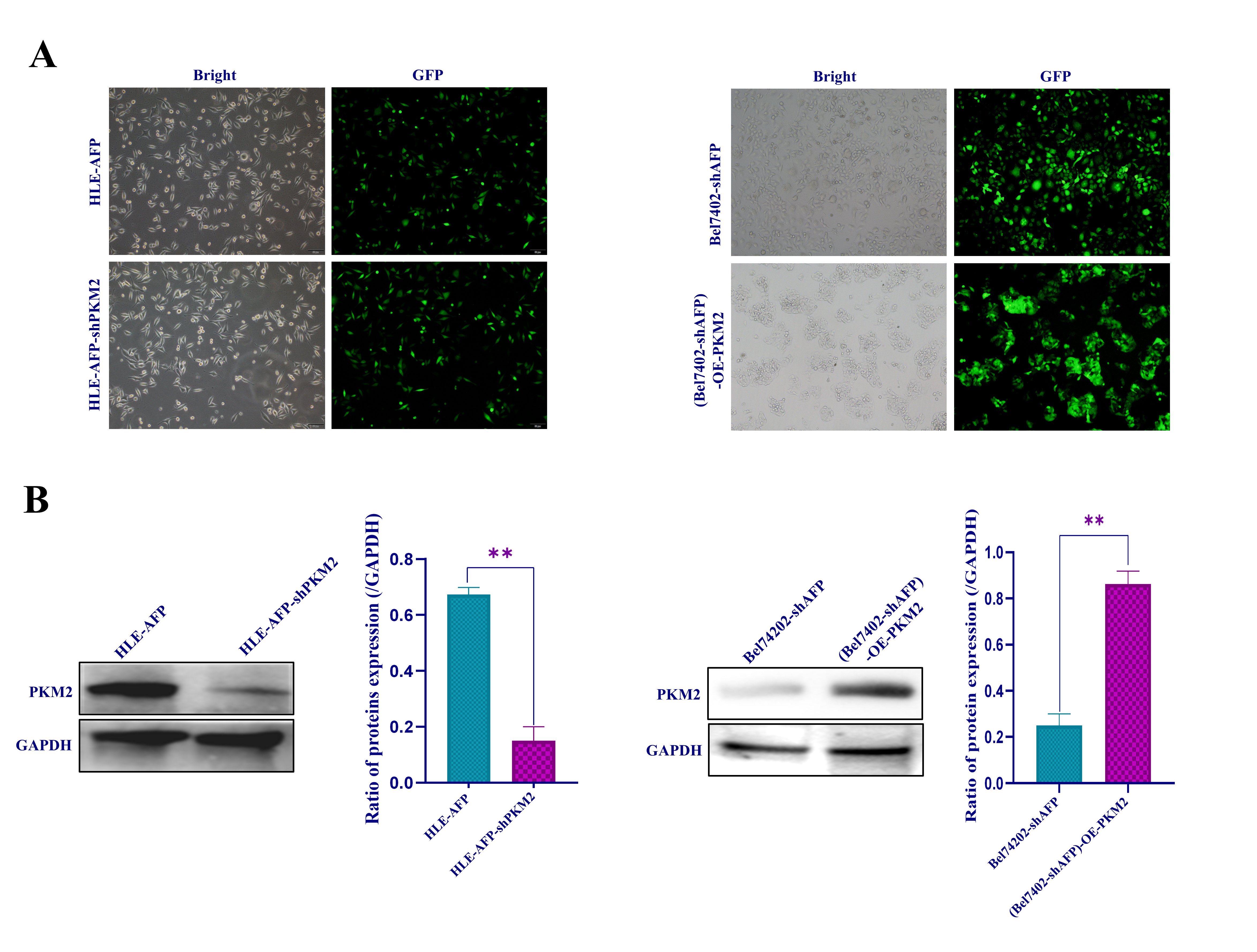

Supplement: Supplementary file 3 — Figure S3: The effect of interference or overexpression vectors on the expression of PKM2 in HCC cells. (A) Transfection efficiency in HLE‐AFP cells or Bel7402‐shAFP cells were observed using a fluorescence microscope. (B) The efficiency of PKM2 interference in HLE‐AFP cells or overexpression in Bel7402‐shAFP cells were verified by Western blotting, the bar chart on the right shows statistical analysis of protein grey scan value. **p < 0.01. The image represents the experiment repeated three times. [file JCMM-30-e71226-s001.tif]

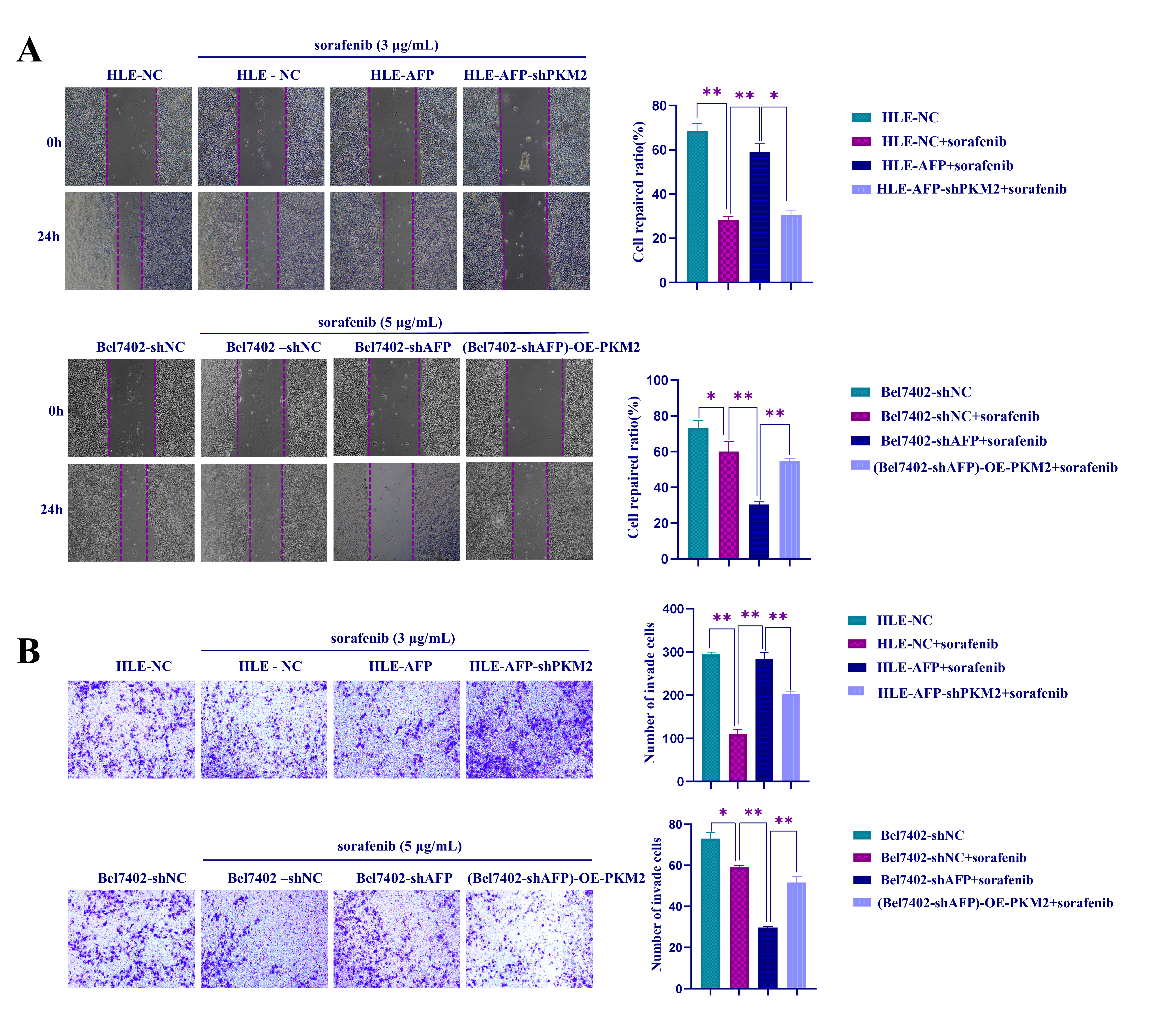

Supplement: Supplementary file 4 — Figure S4: The effect of PKM2 on AFP antagonizing the inhibitory effect of sorafenib on the migration of HCC cells. HLE, HLE‐AFP and HLE‐AFP‐shPKM2 cells were treated with sorafenib (3 μg/mL), or Bel7402, Bel7402‐shAFP and (Bel7402‐shAFP)‐OE‐PKM2 cells were treated with sorafenib (5 μg/mL) for 48 h, and the scratch test (A) and transwell test (B) are used to detect the migratory ability of HCC cells, the bar chart on the right shows statistical analysis. *p < 0.05, **p < 0.01. The picture represents the results of three repeated experiment. [file JCMM-30-e71226-s004.jpg]
